# Gene Expression Profiling to Unfolded Proteins Response as a Risk Modulator of Patients with Rheumatoid Arthritis

**DOI:** 10.3390/ijms25094854

**Published:** 2024-04-29

**Authors:** Aleksandra Kucharska-Lusina, Maciej Skrzypek, Aleksandra Binda, Ireneusz Majsterek

**Affiliations:** Department of Clinical Chemistry and Biochemistry, Medical University of Lodz, 92-215 Lodz, Poland; ola_kucharska@wp.pl (A.K.-L.); maciej.skrzypek@umed.lodz.pl (M.S.); aleksandra.binda@umed.lodz.pl (A.B.)

**Keywords:** endoplasmic reticulum stress, unfolded protein response, ER-adaptosome, PERK inhibitor, rheumatoid arthritis

## Abstract

Rheumatoid arthritis (RA) is a chronic inflammatory disease. Despite new methods of diagnostics and treatment as well as extensive biological and immunosuppressive treatment, the etiology of RA is not fully understood. Moreover, the problem of diagnosis and treatment of RA patients is still current and affects a large group of patients. It is suggested that endoplasmic reticulum (ER)-related features may impair adaptation to chronic stress, inferring the risk of rheumatoid arthritis. The main goal in this study was evaluation of changes in mRNA translation to determine chronic ER stress conditions in rheumatoid arthritis patients. The study group consist of 86 individuals including a total of 56 rheumatoid arthritis patients and 30 healthy controls. The expression level of mRNA form blood samples of RA patients as well as controls of the unfolded protein response (UPR)-associated genes (*p-eIF2*, *BCL-2*, *PERK*, *ATF4*, and *BAX*) were investigated using real-time qPCR. *GAPDH* expression was used as a standard control. Considering the median, the expression levels of *PERK*, *BCL-2*, *p-eIF2*, *ATF4*, and *BAX* were found to be significantly increased in the blood of RA patients compared with the control group. The *p*-value for the *PERK* gene was 0.0000000036, the *p*-value for the *BCL-2* gene was 0.000000014, the *p*-value for the *p-eIF2* gene was 0.006948, the *p*-value for the *ATF4* gene was 0.0000056, and the *p*-value for the *BAX* gene was 0.00019, respectively. Thus, it can be concluded that the targeting of the components of the PERK-dependent UPR signaling pathway via small-molecule PERK inhibitors may contribute to the development of novel, innovative treatment strategies against rheumatoid arthritis.

## 1. Introduction

Rheumatoid arthritis (RA) is a systemic condition affecting the joints and their surrounding tissues. RA presents a significant medical concern, and, to date, constitutes a major therapeutic challenge. Approximately, 1% of the global population suffers from RA, making it the most prevalent chronic autoimmune disorder [1]. Typically, the age of initial symptoms is estimated between 35 to 60. It is characterized by severe joint pain, stiffness, and swelling, and if not treated effectively, it can lead to disability, chronic pain, and distress. Proper treatment for RA alleviates pain, enhances functionality, and improves the overall quality of life.

It is known that rheumatoid arthritis is a chronic inflammatory disease characterized by the abnormal proliferation of synoviocytes, leukocyte infiltration, and angiogenesis. Despite new methods of diagnostics and treatment as well as extensive biological and immunosuppressive treatment, which significantly slow down the course of the disease, the problem of diagnosis and treatment of RA patients is still current and affects a large group of patients. The etiology of RA is not fully understood. It has been suggested that its development is influenced by autoimmune, environmental, and genetic factors. Therefore, a substantial number of RA patients do not achieve remission [2].

Most secretory and transmembrane proteins fold and mature in the endoplasmic reticulum (ER). The flux of proteins entering the ER is dynamic and regulated. In demanding states, the protein load in the ER is increased and must be met by the organelle folding capacity. Adaptation to the load requires quality control mechanisms which monitor the levels of unfolded proteins and prevent their accumulation for risks of aggregation [3]. To prevent misfolded proteins from accumulating in the ER, proteins that fail quality control undergo retro-translocation to the cytosol, where they are ubiquitinated. Misfolded, potentially toxic proteins in the lumen and membrane of the endoplasmic reticulum are eliminated by proteasomes in the cytosol via ER-associated degradation (ERAD). The ERAD process involves the recognition of substrates in the ER lumen and membrane, their translocation to the cytosol, ubiquitination, and delivery to the proteasome for degradation [4]. Therefore, a wide range of misfolded targets require a fast and highly processable mechanism. Once these quality control mechanisms are compromised, or when cells undergo an insult that changes the physiology of the secretory pathway, such as viral infection or in response to disease, the balance between folded and unfolded proteins in the ER is tipped, resulting in the accumulation of misfolded proteins, a state referred to as ER-stress [5].

Eukaryotic cells respond to ER stress by activating a signaling pathway coined the unfolded protein response (UPR). The UPR is a collection of signaling pathways which can resolve ER stress by integrating mRNA translation control with the regulation of gene transcription. If ER stress persists despite the activation of these feedback responses, the UPR will initiate apoptosis [6]. In mammalian cells, the UPR is comprised of three major branches: inositol requiring enzyme 1 (IRE1), protein kinase RNA-like ER kinase (PERK), and activating transcription factor 6 (ATF6), each termed after the ER-transmembrane sensors which gauge the levels of misfolded proteins in the ER lumen and consequently activate their respective downstream signaling cascades. Briefly, upon ER stress, the ATF6 transmembrane sensor travels from the ER to the Golgi, where it is cleaved in a manner that liberates the N-terminus domain of ATF6 (ATF6(N)). ATF6(N) translocates to the nucleus and functions as a transcription factor. PERK is activated by oligomerization, and once activated, phosphorylates the translation initiation factor eIF2α. This reduces translation initiation, leading to a global decrease in protein synthesis. Paradoxically, eIF2α phosphorylation increases the synthesis of select transcripts, some of which contain short overlapping open reading frames in their 5′UTR, such as ATF4, a transcription factor that coordinates transcription of genes that determine cell fate following ER stress [7]. The third UPR sensor, IRE1, is kinase and endonuclease. Once activated it splices the mRNA of the transcription factor XBP1, excising a 26-nucleotide intron. This non-canonical splicing causes a shift in the reading frame, yielding the spliced form of XBP1 (XBP1s) [8,9]. XBP1s is a highly potent transcription factor that increases the levels of a large variety of ER chaperones and induces expansion of the ER [10].

The Kyoto Encyclopedia of Genes and Genomes (KEGG) pathway analysis revealed that the group of genes congruently upregulated during chronic ER stress is enriched in those that encode proteins involved in ER functions, including genes listed in reference to PERK-dependent UPR [11]. ER-adaptosome genes (including 35 genes of the ER protein processing pathway) are known targets of UPR-induced transcription factors, including ATF6, which exhibits protective functions during chronic ER stress [12]. One of these factors, the ER transmembrane glycoprotein wolframin, is a regulator of ER calcium levels, which plays a crucial role in ER homeostasis [13]. Because ATF4 induction requires PERK and is necessary for maximal induction of ATF6 [12], it is suggested that sustained PERK activity during chronic ER stress maintains ER proteostasis in concert with congruently upregulated genes. This, in combination with the effects on the ER-localized translation, suggests that chronic ER stress is tailored to maintain ER function by coordinating the protein load and processing capacity of the ER. Transcripts that are predominantly translated at the ER [13] showed congruent decreases in polysome-association and cytosolic mRNA levels during chronic ER stress. Thus, ER-associated mRNA translation is modulated during chronic ER stress in a PERK-dependent manner.

Recent scientific reports indicate that endoplasmic reticulum stress constitutes a significant etiological factor in various human diseases, including conditions associated with the development of inflammation [14]. Specifically examining the PERK cellular signaling pathway, there is promising potential for understanding the pathological mechanisms related to rheumatoid arthritis. Thus, the current study aims to apply real-time qPCR expression of mRNA (the genes of the UPR) to determine global changes in mRNA translation specific for chronic ER stress conditions in rheumatoid arthritis patients.

## 2. Results

### 2.1. Patient Characteristics

Expression analysis included 56 blood samples from the rheumatoid arthritis group (patients from an outpatient clinic, including 39 women and 17 men). The average age among women was 73 years, while, among men, it was 63 years) and 30 blood samples were taken from the control group. Positive family history of inflammatory joint diseases was present in 21 women and 10 men. All the patients were treated with csDMARDs. Short-term oral glucocorticoid therapy was administered to 27 women and 16 men during the initiation or modification of csDMARDs. The mean erythrocyte sedimentation rate (ESR) before treatment initiation in the female group was 47 mm/h [5–10 mm/h], whereas in the male group it was 64 mm/h. The mean C-reactive protein (CRP) level before treatment initiation in the female group was 31 mg/L [0–5 mg/L], while among men it was 38 mg/L. The mean DAS28 score 3 months after treatment initiation was 2.69 among women and 2.58 among men, indicating the achievement of remission. After treatment initiation, a significant decrease in inflammatory parameters was also observed. In the female group, the mean ESR was 19 mm/h, while, in the male group, it was 12 mm/h. The mean CRP level among women was 6 mg/L, while among men it was 4 mg/L (Table 1).

### 2.2. Expression of Endoplasmic Reticulum Stress Genes

**Figure 1:**
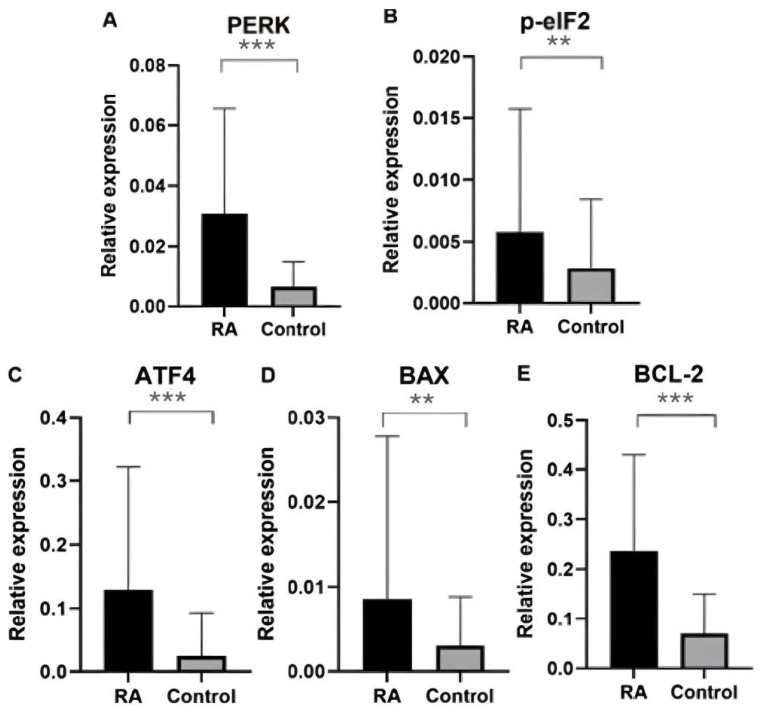
Relative expression of ER stress genes in the blood of rheumatoid arthritis patients and controls: (**A**) *PERK* gene expression in patients and healthy groups; (**B**) *p-eIF2* gene expression in patients and healthy groups; (**C**) *ATF4* gene expression in patients and healthy groups; (**D**) *BAX* gene expression in patients and healthy groups; (**E**) *BCL-2* gene expression in patients and healthy groups. Data were presented as means ± SEM (** *p* < 0.01, and *** *p* < 0.0001).

Gene expression analysis was performed for six genes, including the endogenous control gene *GAPDH* and five test genes, namely *PERK*, *BCL-2*, *p-eIF2*, *ATF4*, and *BAX*. Considering the median, the expression of ER stress genes (*PERK*, *BCL-2*, *p-eIF2*, *ATF4*, and *BAX*) was found to be significantly increased in the blood of RA patients compared with the control group, and the results are presented in Figure 1. The *p*-value for the *PERK* gene was 0.0000000036, the *p*-value for the *BCL-2* gene was 0.000000014, the *p*-value for the *p-eIF2* gene was 0.006948, the *p*-value for the *ATF4* gene was 0.0000056, and the *p*-value for the *BAX* gene was 0.00019, respectively.

**Figure 2:**
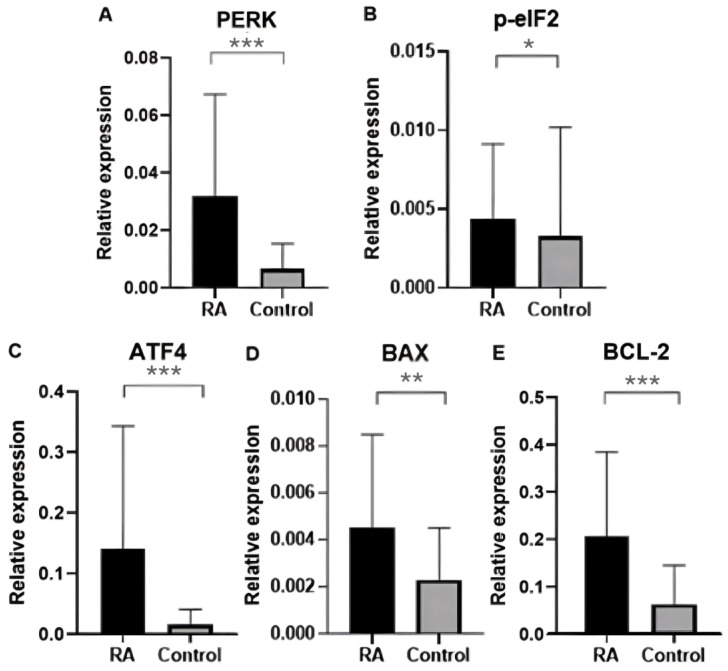
Relative expression of ER stress genes in the blood of rheumatoid arthritis patients and controls in the female group: (**A**) *PERK* gene expression in patients and healthy groups; (**B**) *p-eIF2* gene expression in patients and healthy groups; (**C**) *ATF4* gene expression in patients and healthy groups; (**D**) *BAX* gene expression in patients and healthy groups; (**E**) *BCL-2* gene expression in patients and healthy groups. Data were presented as means ± SEM (* *p* < 0.05, ** *p* < 0.01, and *** *p* < 0.0001).

Additionally, expression was analyzed in blood samples from groups of RA patients and control groups divided by patient gender. In the female group, the expression of ER stress genes (*PERK*, *BCL-2*, *p-eIF2*, *ATF4*, and *BAX*) was found to be significantly increased in the blood of RA patients compared with the control group. The results are shown in Figure 2. The *p*-value for the *PERK* gene was 0.000001026, the *p*-value for the *BCL-2* gene was 0.000004234, the *p*-value for the *p-eIF2* gene was 0.02843, the *p*-value for the *ATF4* gene was 0.00002514 and the *p*-value for the *BAX* gene was 0.002822, respectively.

**Figure 3:**
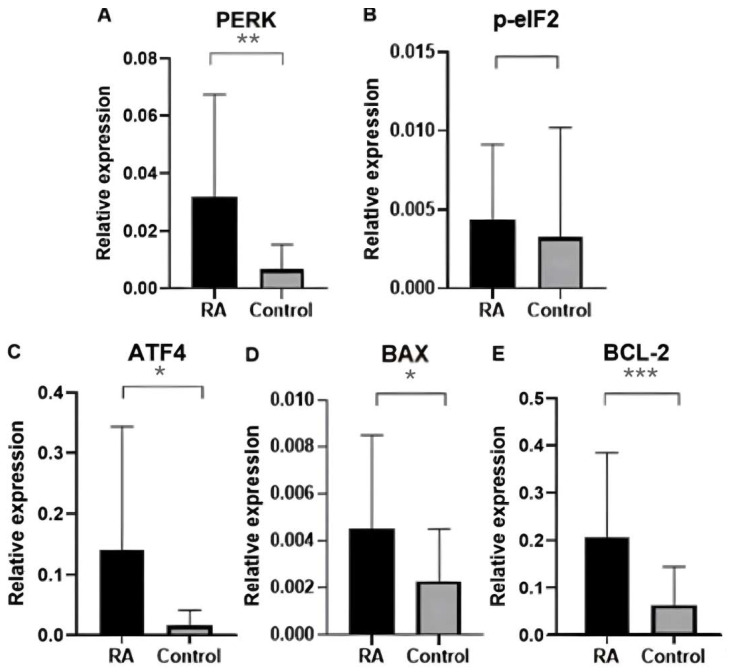
Relative expression of ER stress genes in the blood of rheumatoid arthritis patients and controls in the male group: (**A**) *PERK* gene expression in patients and healthy groups; (**B**) *p-eIF2* gene expression in patients and healthy groups; (**C**) *ATF4* gene expression in patients and healthy groups; (**D**) *BAX* gene expression in patients and healthy groups; E. *BCL-2* gene expression in patients and healthy groups. Data were presented as means ± SEM (* *p* < 0.05, ** *p* < 0.01, and *** *p* < 0.0001).

In the male group, the expression of ER stress genes (*PERK*, *BCL-2*, *ATF4*, and *BAX*) in the blood of RA patients was significantly increased compared with the control group. The *p*-value for the *PERK* gene is 0.0004593. The *p*-value for the *BCL-2* gene is 0.00009458. The *p*-value for the *ATF4* gene is 0.02951. The *p*-value for the *BAX* gene is 0.01845. However, no significant statistical differences were observed for the *p-eIF2* gene (*p* value 0.05535). The results are shown in Figure 3.

Analysis of *PERK*, *BCL-2*, *p-eIF2*, *ATF4*, and *BAX* gene expression between the female and male groups with RA showed no statistically significant differences. The *p*-value for the *PERK* gene was 0.5182.The *p*-value for the *BCL-2* gene was 0.6165, the *p*-value for the *ATF4* gene was 0.2705, the *p*-value for the *BAX* gene was 0.3483 and the *p*-value for the *p-eIF2* gene was 0.5662, respectively.

## 3. Discussion

Rheumatoid arthritis is the most common inflammatory joint disease, affecting approximately 0.3–1.5% of the population. The incidence is 0.1–0.5% in adults. The peak incidence occurs in the fourth and fifth decades of life. Women get sick three times more often than men, and the age of onset for women is usually over 50. Burdensome family history increases the risk of developing the disease many times over. The attempt to classify RA is made using the ACR/EULAR 2010 criteria. The quality of life in RA patients is assessed using the AIMS-2 scale [15].

The etiology of RA is not fully understood, and it has been suggested that its development is influenced by genetic factors. The heredity of rheumatoid arthritis is estimated to be 66%. There is an increased risk of developing rheumatoid arthritis in first-degree relatives. The presence of the disease in a parent increases the risk of its development in a child by 2–5 times [16]. Genome sequencing revealed the association of many genes with an increased risk of disease development [15]. In this study, expression analysis was performed for genes of *PERK*, *BCL-2*, *p-eIF2*, ATF4, and *BAX* in the blood of RA patients compared with healthy individuals.

Endoplasmic reticulum (ER)-associated degradation and the unfolded protein response are two key quality-control machineries in the cell. ERAD is responsible for the clearance of misfolded proteins in the ER for cytosolic proteasomal degradation, while UPR is activated in response to the accumulation of misfolded proteins [3,4,5]. When the balance between folded and unfolded proteins in the ER becomes tipped, resulting in toxic accumulation of misfolded proteins, chronic ER stress begins to dominate abnormal cell function.

Then, eukaryotic cells respond to ER stress and the accumulation of unfolded proteins in the endoplasmic reticulum by activating the unfolded protein response (UPR) [6]. One of the transducers of the mammalian UPR is PERK kinase, which upon ER stress causes the global attenuation of protein synthesis mediated by the phosphorylation of eIF2α. However, protein synthesis largely recovers while stress ensues, indicating an adaptation process. A group of genes, termed the ER-adaptosome, was induced transcriptionally and escaped translation repression under chronic ER stress conditions [7]. Eukaryotic cells with developed ER express different ER-translated mRNAs than normal cells [17]. It is suggested that these ER-related features may impair adaptation to chronic stress, inferring the risk of rheumatoid arthritis.

In addition to its roles in development and cell function, the UPR modulates prominent diseases, such as diabetes, liver steatosis, inflammatory bowel disease, cancers, and more [18,19,20,21]. The role of ER stress in rheumatoid arthritis was initially suggested [22,23]. However, the involvement of the UPR in RA patients has turned out to be much more general, with important contributions to disease initiation, progression, and response to therapy [24,25]. In agreement with the PERK-mediated maintenance of the adaptive state during chronic stress conditions, the inclusion of PERK inhibitors resulted in the formation of large membrane distensions, associated with perturbation of the ER-adaptosome. These intracellular distensions gradually ballooned, and the foamy cells eventually died in a manner that coincided with the rupture of the vacuoles [26]. However, in agreement with the idea that intracellular ballooning is a sign of ER dysfunction and not a programmed cell death was the finding that the vacuoles were reversed back to normal ER structures and perfectly functioning cells if the stress was removed shortly after the appearance of ER distension [27]. The ER-dysfunction-mediated cell death mechanism is named as ballooning endoplasmic reticulum cell death (BERD). Thus, how the adaptation to chronic ER stress is modulated in rheumatoid arthritis cells and whether a failure to adapt can be used for BERD-mediated treatment of patients with rheumatoid arthritis should be the subject of future studies, to determine the unfolded protein response pathway as a modulator of rheumatoid arthritis initiation, progression, and therapy.

The cellular program controlled by PERK in mouse embryonic fibroblasts (MEFs), represented in normal cells, is widespread and includes many downstream genes regulated by multiple mechanisms [28]. However, PERK affects its targets in a cell-type- and physiological context-dependent manner [29]. For instance, PERK has been found to be essential to the progression of BRAF-mutated melanoma but has less of a role in non-BRAF mutated tumors [30]. PERK regulates cellular redox by directly phosphorylating and activating NRF2 [31,32]. PERK interphases with the circadian oscillations via the induction of miRNA, which represses major circadian genes in a manner that affects Burkitt lymphoma progression [26]. Hence, it is not surprising that multiple pharmaceutical companies developed high-affinity inhibitors of PERK. Glaxo Smith Kline developed GSK2606414 (GSK414); Amgen developed AMG PERK 44; Eli Lilly developed Ly4. In our laboratory we developed specific inhibitors for PERK treatment in neurodegenerative disorders including glaucoma (termed PERKi) [33,34,35,36]. The intricacies of the transcription and translation program controlled by PERK invites research into different tissue types and into combinations with other drugs to assess its role as an efficacious therapeutic target.

Translation repression by PERK in response to chronic ER stress is reversed by adaptation. The phosphorylation of eIF2α by PERK and by additional kinases attenuates translation initiation by the sequestration of the multi-subunit GEF eIF2B. This results in the reduction in the ternary complex of the translation initiation and leads to a global repression in protein synthesis [37]. Since cells cannot survive under prolonged translation repression, homeostatic mechanisms are engaged to gradually restore protein synthesis during chronic ER stress. One such mechanism, described by Hatzoglou et al., is a conversion from the classical and efficient CAP-dependent mRNA translation, driven by eIF4E, to a less favorable mechanism that relies on recruitment of the ribosome by eIF3 [38]. This adaptation process is dependent on the constant repression of eIF2B activity [39]. Analysis of the stress-specific transcriptome and translatome in MEFs subjected to a prolonged ER stress identified a set of 567 genes that were induced at the level of transcription and were translated under the chronic conditions [17]. Remarkably, 35 of these genes encode proteins that function in the ER in protein folding, glycosylation, trafficking, and degradation [12]. Genes were identified under ER stress conditions using thapsigargin (Tg:16h vs. Tg:1h). Because the ER protein processing pathway includes, in total, approximately 141 genes (according to KEGG, PATHWAY: ko04141 in www.genome.jp (accessed on 11 September 2014)), it is of note that a relatively large subset of genes specifically in this category were upregulated during adaptation to ER chronic stress [13]. When PERKi was applied post-establishment of the transcriptional and translational reprogramming, this led to a 50% larger shift in fold changes for ER-translated mRNAs, which was determined by the comparison of polysome-associated- to total mRNAs. Consistently, these mRNAs were regulated via both changes in translation efficiency (61 genes) and mRNA abundance (105 genes) [17]. The term ER-adaptosome was proposed to describe the group of genes congruently induced during chronic ER stress.

The main gene that has been implicated in the pathogenesis of RA is the *PERK* gene, which is involved in the unfolded protein response (UPR) pathway [22]. Studies have shown that *PERK* gene expression is upregulated in RA patients, leading to increased activation of the UPR pathway [23]. This chronic ER stress can contribute to the development and progression of RA by inducing inflammatory cells to release cytokines, further perpetuating the inflammatory response [22]. Given the role of PERK in RA pathogenesis, researchers have explored the use of PERK inhibitors as a potential treatment option. Inhibition of PERK has been shown to decrease the production of inflammatory cytokines and to reduce joint inflammation in animal models of RA [40]. Additionally, elevated expression of autophagy-related genes, including Beclin-1, has been observed in RA patients, suggesting a potential link between PERK-mediated ER stress and autophagy in disease pathogenesis [41]. Moreover, the administration of GRP78/BiP, a protein involved in the UPR pathway, has been shown to have potential therapeutic benefits in RA [22]. However, more research is needed to fully understand the complex relationship between *PERK* gene expression and RA.

It was also demonstrated that the phosphorylation of eukaryotic initiation factor 2 (*p-eIF2*) might be a critical event in the regulation of protein synthesis and might play a crucial role in the inflammatory response [42]. In RA, *p-eIF2* gene expression has been shown to be involved in the inflammatory and proliferative processes of the disease [42]. Research studies have demonstrated that *p-eIF2* gene expression is upregulated in RA synovial fibroblasts, which are the cells that line the joints and contribute to the inflammation and destruction of joint tissue [43]. Several research studies have investigated the correlation between RA and *p-eIF2* gene expression. One study found that established RA patients display differentially expressed genes coding for cytokine/chemokine-mediated immunity compared to healthy individuals [44]. Another study demonstrated that cleaved ATF6, a protein that regulates *p-eIF2* gene expression, increases the expression of genes associated with inflammatory responses in RA [45].

Another gene that has been implicated in the development and progression of RA is the activating transcription factor 4 (ATF4) [46]. ATF4 is a transcription factor that is involved in the regulation of cellular stress responses and the maintenance of cellular homeostasis [47]. It is expressed constitutively at low concentrations but can be rapidly induced under certain stress conditions [48]. Research findings have provided insights into the relationship between *ATF4* gene expression and RA. In study conducted on gene expression in RA, ATF4 was upregulated in the synovial membrane [49]. These findings suggest that ATF4 may be involved in the pathogenesis of RA and may serve as a potential biomarker for the disease.

While the exact cause of RA is unknown, genetic and environmental factors are believed to play a role in its development. Thus, recent research has focused on the role of apoptosis, or programmed cell death, in the pathogenesis of RA, and the BAX gene has emerged as a potential contributor to the disease. BAX is a pro-apoptotic gene that may play a critical role in regulating cell death in many different autoimmune diseases [50,51]. The aim of the research by Hillber et al. was an analysis of the expression of apoptosis-related molecules in the synovium of rheumatoid arthritis patients, and the BAX apoptosis accelerator was higher than in healthy controls [52].

This imbalance in apoptosis-related molecules may contribute to the survival of autoreactive lymphocytes, leading to chronic inflammation in the synovial membrane. In a study conducted by Isomäki et al., the expression of the anti-apoptotic gene BCL-2 was studied in peripheral blood, synovial fluid lymphocytes, and synovial tissues from patients with RA [53]. The results indicated that the expression of BCL-2 was not increased in the lymphocytes or synovial tissues derived from patients with RA. Instead, decreased expression of BCL-2 was observed, suggesting that impaired apoptosis may contribute to the pathogenesis of RA [53]. However, another study found that interleukin-17 upregulates the expression of BCL-2 in synoviocytes in RA, indicating a potential role for BCL-2 in the inflammatory response in RA [54]. Overall, the role of *BCL-2* gene expression in RA remains unclear and requires further investigation in order to fully understand its potential contribution to the disease. Although endoplasmic reticulum (ER)-associated degradation and the unfolded protein response are two key quality-control machineries in the cell, it is suggested that BCL-2 is connected to ERAD protein synthesis regulation, but upregulation of BAX gene may be associated with UPR activation in response to the accumulation of misfolded proteins during chronic inflammation in RA patients [5].

Rheumatoid arthritis is an autoimmune disorder characterized by chronic inflammation of the joints, leading to pain, stiffness, and swelling [1]. While the exact cause of RA is unknown, research has shown that genetic factors play a significant role in disease development [2]. In this study, the gene expression of the ER proteins processing pathway was analyzed in blood samples of rheumatoid arthritis (RA) patients in comparison to control group subjects. Gene expression analysis was performed for *PERK, BCL-2, p-eIF2, ATF4,* and *BAX*, as well as the endogenous control gene *GAPDH*. Regarding the literature data, the expression of ER stress genes (*PERK, BCL-2, p-eIF2, ATF4,* and *BAX*) was found to be significantly higher in RA patients than in the control group. While the exact cause of RA is unknown, the present research shows that genetic factors might play a significant role in disease development. According to the literature data, the disease affects the global population, but it is more common in women than men [2]. Interestingly, the research revealed that PERK-related UPR genes in patients divided by gender were found to be higher compared to controls in both the male and female groups, which suggested that the unfolded proteins response is a global process involved in RA pathogenesis. Finally, the exact cause of RA is not fully understood, but it is suggested to involve the UPR’s complex interplay between genetic and environmental factors. While the present findings are promising, further research is needed to determine the safety and efficacy of PERK inhibitors as a treatment for RA in patients.

## 4. Materials and Methods

### 4.1. Patients and Study Specimens

Overall, the research encompassed a group of 86 individuals. The study group comprised 56 patients with diagnosed rheumatoid arthritis, selected from patients of the Vadimed Medical Center in Krakow, Poland. The patients were recruited randomly between 2021–2023. Concurrently, the control group included 30 volunteers selected from healthy subjects admitted to the Medical Center for other reasons (patients admitted for routine dental check-up or prophylactic cervical cytology) not associated with chronic inflammatory, cancer, nor neurodegenerative disorders. All stages (blood sampling for genetics, acute phase reactants, and clinical examination) took place in the same day. The control group was matched to the study group regarding sex and age. Blood samples were collected from both groups for gene expression assessment. The study received approval from the Institutional Bioethics Committee (protocol no. 7/KBL/OIL/2022). All participants provided written informed consent to participate in the study. Prior to commencing the experiments, all participants underwent comprehensive medical examinations.

The study group of patients included a total 56 rheumatoid arthritis patients, including 39 women and 17 men. Study participants were patients from the outpatient clinic with newly diagnosed (period of time from the first outpatient visit to the diagnosis of RA was up to a month) rheumatoid arthritis by one rheumatologist. The diagnoses also fulfilled the ACR/EULAR criteria [55]. Inclusion criteria: diagnosis of rheumatoid arthritis based on established diagnostic criteria, age > 45, stable health status, ability to provide informed consent, willingness to follow research procedures: taking medications regularly. Exclusion criteria: age < 45, presence of other autoimmune diseases, recent use of immunosuppressivse medications, inability to provide informed consent, unwillingness to follow research procedures. The Disease Activity Score (DAS28) was also analyzed, which is commonly used in clinical practice to assess disease activity and joint damage [56]. The mean DAS28 score before treatment initiation in the female group was 5.76, while in the male group it was 6.23. All patients were naïve to treatment and were treated according to current ACR/EULAR recommendations [57].

### 4.2. RNA Isolation

Total RNA was extracted from whole blood in a sterile environment using a commercially available RiboPure™-Blood Kit (Invitrogen™, ThermoFisher Scientific, Waltham, MA, USA) in accordance with the manufacturer’s protocol. The concentration and quality of the extracted RNA were evaluated by spectrophotometric measurement of samples at 260 and 280 nm using a Multiskan SkyHigh Microplate Spectrophotometer (Thermo Scientific™, ThermoFisher Scientific, Waltham, MA, USA).

### 4.3. Generation of Single-Stranded cDNA

Obtained RNA was used for the quantitative conversion of 100 ng of total RNA into cDNA in single 10 μL reaction using the High-Capacity cDNA Reverse Transcription Kit (Applied Biosystems™, ThermoFisher Scientific, Waltham, MA, USA) in accordance with the manufacturer’s instructions. To perform reverse transcription, the thermal cycler VeritiPro (Applied Biosystems™, ThermoFisher Scientific, Waltham, MA, USA) was used, according to the conditions suggested by the manufacturer.

### 4.4. Quantitative Real-Time PCR (qPCR)

The expression of genes associated with the selected *PERK* signaling pathway in the study and control groups was evaluated using the TaqMan technique. For qPCR reactions, 10 ng of generated cDNA was used for analysis on the CFX Connect Real-Time PCR Detection System (Bio-Rad, Hercules, CA, USA). Quantification of mRNA expression was performed using the TaqMan™ gene expression assays, including TaqMan Universal PCR Master Mix (Applied Biosystems™, ThermoFisher Scientific, Waltham, MA, USA) with the predeveloped TaqMan assays for *EIF2A* (Assay ID HS00230684_m1), *BCL-2* (Assay ID HS00708019_s1), *PERK* (Assay ID HS00984003_n1), *p-eIF2* (Assay ID Hs00909569_g1), *BAX* (Assay ID Hs00180269_m1), and *GAPDH* (Assay ID HS02786624_g1). *GAPDH* expression was used as an endogenous standard. The targeted transcripts were run in triplicate. Real-time PCR conditions were as universal cycling conditions in accordance with standard protocol. The quantities of selected genes in relation to the housekeeping gene were assessed using the comparative Ct method by Livak.

### 4.5. Statistical Analysis

Statistical analyses were performed using Statistica 13.1 software (StatSoft, Tulsa, OK, USA). Data on clinical characteristic of patients with rheumatoid arthritis were presented as means ± SD (standard deviation of the mean). Data on the expression analysis of genes were presented as means ± SEM (standard error of the mean) of the conducted experiments. For the expression analysis, the distribution of variables was evaluated using the Shapiro–Wilk test, and statistical analysis of differences between the groups of data was carried out using the Mann–Whitney U test (for non-normal distribution). Values of *p* < 0.05 were regarded as statistically significant (* *p* < 0.05, ** *p* < 0.01, and *** *p* < 0.0001).

## 5. Conclusions

In conclusion, based on the latest literature data and results obtained from the present study, gene expression profiling for the unfolded proteins response can be considered as a risk modulator of patients with rheumatoid arthritis. Finally, it can be concluded that targeting of the components of the PERK-dependent UPR signaling pathway via small-molecule PERK inhibitors, may contribute to the development of novel, innovative treatment strategies against rheumatoid arthritis.

## Figures and Tables

**Table 1:** Clinical characteristics of rheumatoid arthritis patients.

Category	Mean Value	SD
Number of patients (n)	56	
Number of patients treated with methotrexate (MTX) (n)	50	
Positive family history (n)	31	
Number of patients bridging with GCS (n)	43	
Mean ESR before treatment initiation (mm/h)	55.5	27.3
Mean ESR after treatment initiation (mm/h)	15.5	14.2
Mean CRP before treatment initiation (mg/L)	34.5	33.4
Mean CRP after treatment initiation (mg/L)	5	6.3
Mean DAS-28 before treatment	5.99	1.51
Mean DAS-28 after treatment	2.63	0.7

Data were presented as means ± SD.

## Data Availability

The data that support the findings of this study are available from the corresponding author upon reasonable request.
